# Robotic versus laparoscopic right hemicolectomy: a retrospective single-center comparative cohort study

**DOI:** 10.1007/s11701-026-04070-5

**Published:** 2026-10-26

**Authors:** Duno Molinari, Felicitas Wiedemann, Marco von Strauss, Fiorenzo V. Angehrn, Beat P. Müller, Daniel C. Steinemann, Fabio Nocera

**Affiliations:** 1 Department of Surgery, Clarunis - University Digestive Health Care Center Basel - Clarunis, Site St. Claraspital, Kleinriehenstrasse 30, 4058 Basel, Switzerland; 2 Medical Faculty, University of Basel, Basel, Switzerland

**Keywords:** Robotic surgery, Laparoscopic surgery, Right hemicolectomy, Colorectal surgery

## Abstract

We compared perioperative outcomes after robotic (rRC) and laparoscopic right hemicolectomy (lapRC) and assessed temporal changes in surgical approach and minimally invasive completion. This retrospective cohort included patients operated between January 2023 and March 2026. Primary outcomes were operative duration, open conversion, and 30-day morbidity. The main comparison was unadjusted, supplemented by elective and propensity-score-matched analyses. Temporal trends included all screened patients, including primary open procedures. A total of 221 patients were analyzed, including 122 rRC and 99 lapRC procedures. Median operative duration was greater with rRC than with lapRC (264 vs. 200 min, p < 0.001), whereas conversion to an open procedure occurred less frequently after rRC (4% vs. 20%, p < 0.001). Overall morbidity (28% vs. 35%, p = 0.246) and major complications (12% vs. 12%, p = 1) did not differ significantly. Median lymph-node yield in carcinoma resections was 26 versus 25 (p = 0.249). The conversion difference was not statistically significant in the overall elective cohort or the matched analysis of 32 pairs. Across all 390 screened patients, increasing robotic use coincided with decreasing primary open surgery (p = 0.014), while the increase in minimally invasive completion was not statistically significant (p = 0.075). Robotic right hemicolectomy may offer an advantage in reducing conversion to open surgery, although operative duration was longer. This potential benefit requires confirmation, given baseline differences and the limited size of the matched cohort.

**Supplementary Information:**

The online version contains supplementary material available at https://doi.org/10.1007/s11701-026-04070-5.

## Introduction

Minimally invasive approaches have progressively gained prominence in colorectal surgery over the past two decades. For right hemicolectomy, laparoscopy has been associated with more favorable perioperative outcomes than the conventional open approach [1]. Reported benefits include reduced postoperative morbidity and faster recovery [1], while oncological outcomes remain comparable [2].

From an oncological perspective, complete mesocolic excision (CME) with central vascular ligation aims to achieve an oncologically radical resection, following principles similar to total mesorectal excision (TME) in rectal cancer [3, 4]. Nevertheless, CME during right hemicolectomy can be technically demanding. In particular, dissection around the central vascular structures is complicated by the close proximity of the superior mesenteric vessels and other adjacent anatomical structures. The RELARC randomized trial, which compared laparoscopic CME with D2 dissection in right hemicolectomy, reported a higher incidence of vascular injury with CME (3.0% vs. 1.2%, p = 0.045) [5, 6]. These findings highlight the technical challenges of central vascular dissection in this anatomically complex region.

Robotic surgery may offer technical advantages in this setting, as it combines three-dimensional visualization with articulated instruments, a stable camera platform, tremor reduction, and improved surgeon ergonomics [7, 8]. These technological features may facilitate dissection around the central mesenteric vessels and support the minimally invasive performance of CME.

Despite existing comparisons, the relationship between increasing robotic use and overall minimally invasive completion remains unclear. We compared perioperative outcomes after robotic and laparoscopic right hemicolectomy and assessed temporal trends across all screened patients. Our exploratory hypothesis was that robotic surgery would be associated with fewer conversions and increasing robotic use with a higher overall rate of minimally invasive completion.

## Methods

### Patients

All individuals treated with a right or extended right hemicolectomy at Clarunis – University Digestive Health Care Center, Basel, Switzerland, from January 2023 through March 2026 were screened for this retrospective single-center cohort study. The study period corresponded to the start of systematic data collection in a prospectively maintained colorectal database. Institutional general consent permitting the use of clinical data for research had been provided by all patients included in the analysis. Valid signed consent and the absence of a documented withdrawal were verified for each included patient.

Eligibility required patients to be at least 18 years of age, to have undergone a robotic or laparoscopic right or extended right hemicolectomy, and to have received a primary intestinal anastomosis during the index operation. Patients were excluded if surgery was performed through a primary open approach, if no primary anastomosis was created, or if major multivisceral resection was required.

Patients were grouped according to the initially attempted surgical approach into a robotic right hemicolectomy cohort (rRC) and a laparoscopic cohort (lapRC). Patients converted to open surgery remained assigned to their initial surgical approach. A separate analysis of the full screened series, including primary open procedures, assessed temporal changes in surgical approach and minimally invasive completion.

### Data collection

Clinical and operative data were obtained from the prospectively maintained electronic database and managed in REDCap (Research Electronic Data Capture) at University Hospital Basel [9]. Baseline variables included age, sex, body mass index (BMI), American Society of Anesthesiologists (ASA) classification, surgical indication, bowel perforation, and emergency presentation.

Operative variables included the type of resection (right or extended right hemicolectomy), surgical technique, operative time, conversion and its underlying reason, stoma formation, and teaching status. Operative time was defined as the interval from the first skin incision to completion of skin closure, including docking for robotic procedures. Docking time was not recorded separately. Conversion was defined as an unplanned switch to open laparotomy for open continuation of the operation; enlargement of the specimen extraction incision alone was not classified as conversion. The recorded surgeon was the surgeon performing the operation rather than the supervising surgeon. Teaching procedures involved performance of substantial operative steps by a trainee under supervision.

Postoperative assessment covered hospital stay, 30-day reoperation and readmission, and mortality. All postoperative complications and mortality were assessed within 30 days of surgery by retrospective review of institutional clinical records, including documented readmissions. Events occurring at other institutions were captured only if documented in these records. Complications were classified according to Clavien–Dindo [10], with events of grade III or above categorized as major morbidity. The Comprehensive Complication Index (CCI) [11], incorporating all postoperative complications documented within 30 days, was additionally determined for each patient.

For patients undergoing surgery for malignant disease, oncological and pathological variables included neoadjuvant chemotherapy, tumor location, histopathological grade (G-status), the numbers of retrieved and metastatic lymph nodes, clinical and pathological TNM categories, and resection status (R0). The pathologist’s qualitative assessment of CME quality was recorded where available, although no standardized mesocolic-plane grading was performed. Cases without a documented assessment were classified as having missing CME quality data. Specimen length was not available.

Operative time, conversion to open surgery, and postoperative morbidity were defined as the primary outcomes, while the remaining postoperative and oncological variables were considered secondary outcomes.

### Surgical technique

For laparoscopic right hemicolectomy, patients were positioned supine. Pneumoperitoneum was established using a Veress needle at Palmer’s point. A 12-mm umbilical optical trocar, a suprapubic 5-mm trocar, and a left lower quadrant 12-mm trocar were inserted, with an additional right-sided 5-mm trocar when required. Dissection proceeded anterior to Gerota’s fascia, preserving the right ureter and protecting the duodenum and pancreatic head. For malignant disease, CME followed embryological planes with preservation of the mesocolic fascia and central vascular ligation. The superior mesenteric vein (SMV) was visualized, and the ileocolic pedicle was secured with Hem-o-lok® clips before division. Following mobilization, specimens were extracted through a small midline or right upper quadrant incision according to surgeon preference. An ileotransverse anastomosis was created extracorporeally by hand sewing or stapling.

Robotic procedures used the da Vinci Xi® dual-console system with four 8-mm trocars in a linear infraumbilical configuration, spaced approximately 6–8 cm apart. Dissection followed the same medial-to-lateral concept. For malignant disease, CME included SMV exposure and central division of the ileocolic vessels and, where indicated by tumour location or vascular anatomy, the right or middle colic vessels, with accompanying lymphadenectomy. In procedures completed robotically, an intracorporeal ileotransverse anastomosis was created using a robotic stapler with a green reload. The enterotomy was closed with two Stratafix™ barbed sutures in a continuous double-row technique. Specimens were extracted through a Pfannenstiel incision. Mesenteric defects were closed in both groups.

Laparoscopic procedures were performed by 29 surgeons and robotic procedures by 14 surgeons. Experienced surgeons in the laparoscopic group had decades of experience in laparoscopic surgery across the range of visceral procedures. Robotic surgery was introduced at our institution in 2015, although further learning and refinement of robotic techniques may have continued during the study period. Less experienced surgeons always operated under the supervision of experienced surgeons.

### Statistical analysis

Continuous variables were reported as median with interquartile range (IQR) and compared using the Mann–Whitney U test. Categorical variables were reported as counts and percentages and compared using Fisher’s exact test. Variables with more than two categories were assessed using the Fisher–Freeman–Halton test with 100,000 Monte Carlo samples. Conversion reasons were summarized descriptively. Missing values were not imputed.

The main analysis included all patients in the robotic and laparoscopic comparative cohort. Sensitivity analyses were restricted to elective procedures and elective oncological resections, including carcinomas and neuroendocrine tumors. Carcinoma-specific pathological analyses excluded neuroendocrine tumors.

Elective patients were additionally matched 1:1 without replacement using propensity scores estimated by logistic regression from age, sex, BMI, ASA group (I/II, III, IV), indication (carcinoma, neuroendocrine tumor, other), and surgery date. Clinical T-stage was omitted because of limited availability. Matching required exact agreement on sex, ASA group, and indication, within a caliper of 0.2 standard deviations of the propensity-score logit. The algorithm maximized the number of pairs and then minimized total Mahalanobis distance across baseline covariates. Balance was assessed using standardized differences with the unmatched elective cohort’s standardizing denominator; absolute values below 0.10 indicated adequate balance. Matched outcomes were compared using exact McNemar and Wilcoxon signed-rank tests.

Temporal trends were assessed against surgery date using unadjusted linear regression with HC3 robust standard errors for operative duration and logistic regression for robotic use, primary open surgery, and minimally invasive completion. The latter was defined as completion of an initially minimally invasive procedure without open conversion.

A two-sided p value < 0.05 was considered statistically significant. Additional analyses were exploratory, without adjustment for multiple comparisons. All analyses were performed using Python 3.12.14, NumPy 2.3.5, and SciPy 1.17.0 [12, 13].

## Results

From January 2023 to March 2026, 390 patients underwent right or extended right hemicolectomy at our institution for benign or malignant disease. Of these, 155 underwent a primary open procedure and were excluded from the comparative cohort, leaving 235 patients who were initially treated using a minimally invasive approach.

**Fig. 1:**
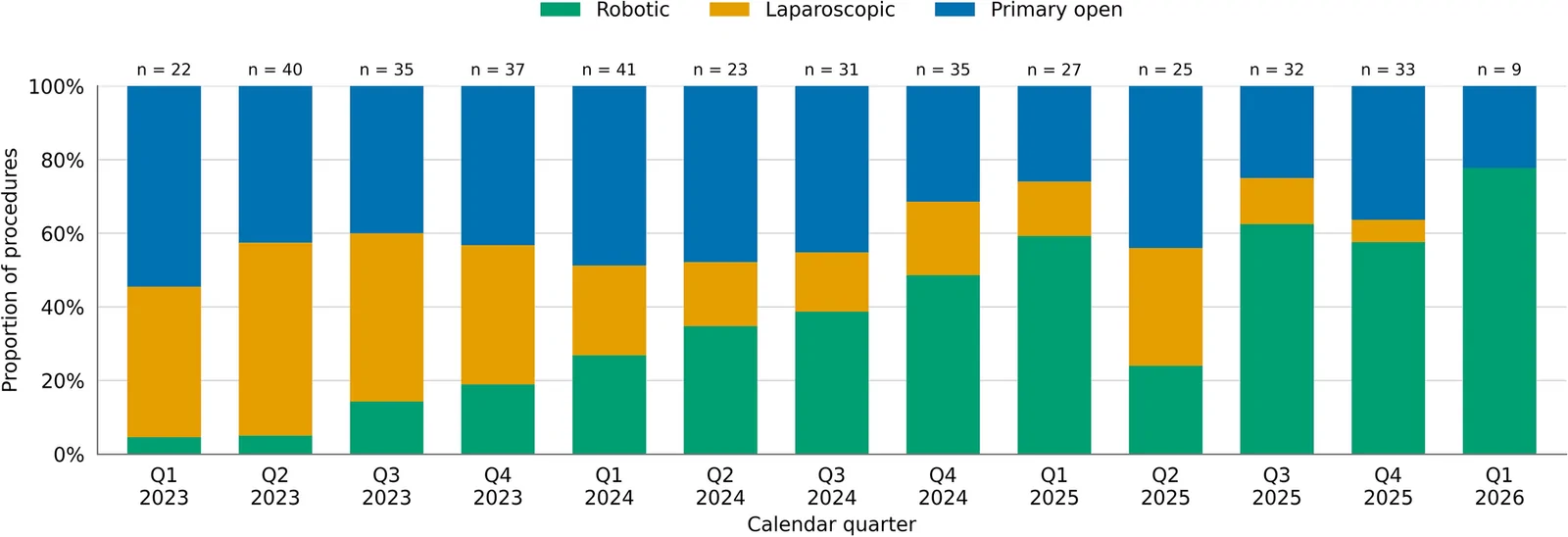
Initial surgical approach by quarter (2023–2026; n = 390). Numbers above bars indicate quarterly case counts

Across the full screened series, the proportion of robotic procedures increased from 11.2% in 2023 to 52.1% in 2025, while primary open procedures decreased from 44.0% to 32.5%. During the same period, the proportion of procedures completed minimally invasively increased from 47.0% to 55.6%. Unadjusted regression against surgery date showed increasing robotic use (p < 0.001) and decreasing primary open surgery (p = 0.014). The increase in minimally invasive completion did not reach statistical significance (odds ratio per year 1.23, 95% CI 0.98–1.55; p = 0.075). These trend analyses included the entire study period. Of the nine procedures recorded in 2026, seven were started robotically and six were completed minimally invasively. The temporal distribution of surgical approaches is shown in Fig. 1.

**Fig. 2:**
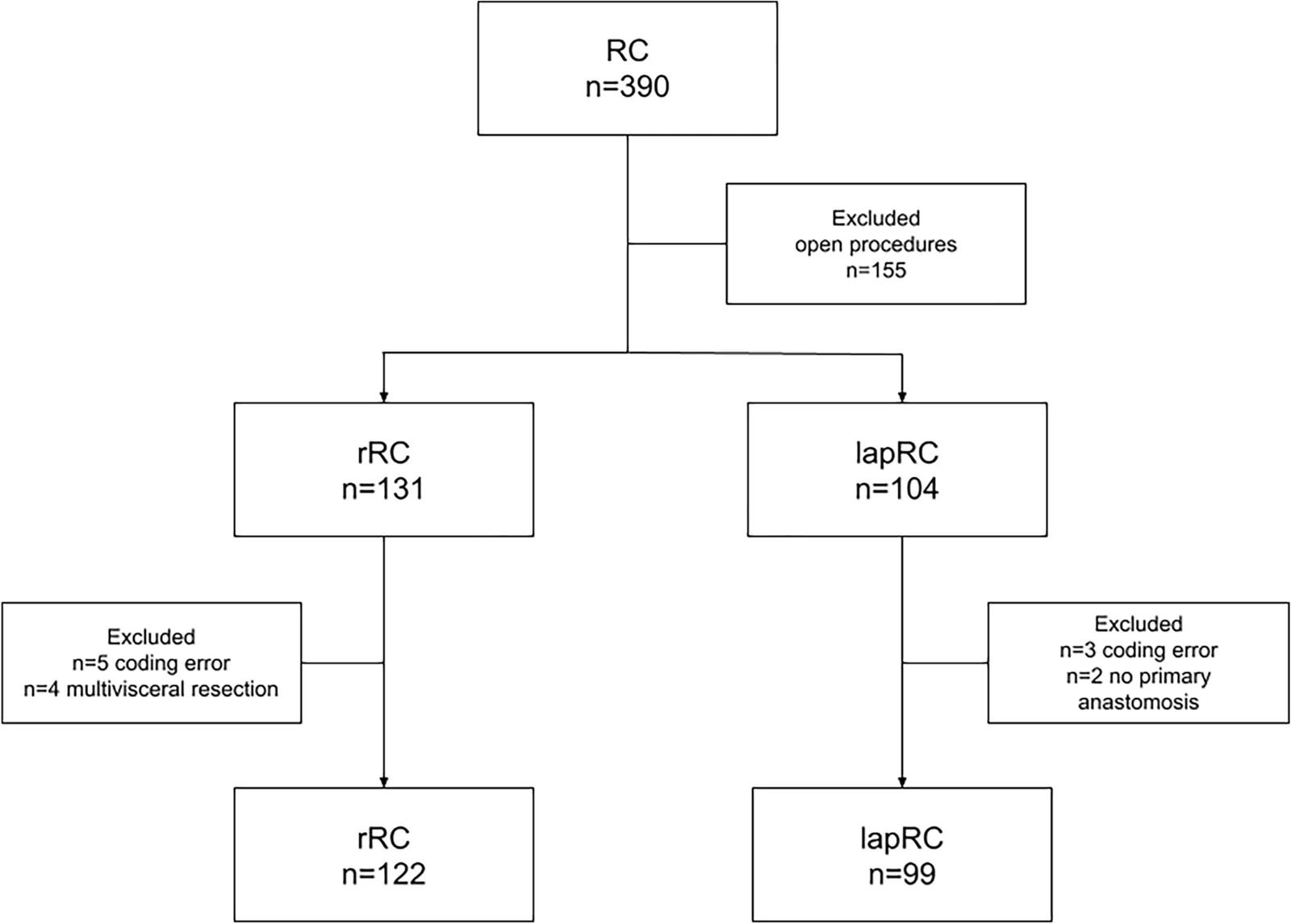
Flow chart of patient screening, exclusion, and allocation to rRC and lapRC cohorts. RC=right hemicolectomy, rRC=robotic right hemicolectomy, lapRC=laparoscopic right hemicolectomy

Following application of the remaining eligibility criteria, 221 patients constituted the study cohort. The final cohort included 122 patients treated robotically and 99 treated laparoscopically; patient selection is shown in Fig. 2.

### Patient characteristics

Patient characteristics are reported in Table 1. Age, sex, and BMI were similar between the two cohorts. Median age was 75 years (IQR 62–83) following rRC and 73 years (IQR 63–78) following lapRC (p = 0.145). Median BMI was 25.0 kg/m^2^ (IQR 22–28) and 24.9 kg/m^2^ (IQR 21.8–29), respectively (p = 0.746). Men represented 51% of the rRC cohort and 52% of the lapRC cohort (p = 1).

**Table 1:** Demographic and clinical characteristics of patients in robotic and laparoscopic right hemicolectomy groups

	rRC n=122	lapRC n=99	p-value
Age, years, median (IQR)	75 (62–83)	73 (63–78)	0.145
Sex, n (%) Male Female	62 (51) 60 (49)	51 (52) 48 (48)	1
BMI, kg/m^2^, median (IQR)	25.0 (22–28)	24.9 (21.8–29)	0.746
ASA classification, n (%) ASA I ASA II ASA III ASA IV	0 (0) 23 (19) 84 (69) 15 (12)	1 (1) 35 (35) 53 (54) 10 (10)	0.018
Emergency surgery, n (%) Yes No	2 (2) 120 (98)	20 (20) 79 (80)	<0.001
Surgical indication, n (%) Carcinoma NET Benign lesion Bleeding Perforation IBD Volvulus	103 (84) 8 (7) 10 (8) 0 (0) 0 (0) 1 (1) 0 (0)	62 (63) 4 (4) 15 (15) 1 (1) 11 (11) 3 (3) 3 (3)	<0.001

Values are median (IQR) or n (%). P values for ASA and surgical indication refer to overall distributions. Benign lesions include polyps and other benign lesions. ASA, American Society of Anesthesiologists; BMI, body mass index; IBD, inflammatory bowel disease; IQR, interquartile range; NET, neuroendocrine tumor; rRC, robotic right hemicolectomy; lapRC, laparoscopic right hemicolectomy

Differences were present in ASA classification and operative indication. The overall ASA distribution differed between groups (p = 0.018). ASA III patients accounted for 69% of the rRC group and 54% of the lapRC group, whereas ASA II was recorded in 19% and 35%, respectively. The distribution of surgical indications also differed between groups (overall p < 0.001). Carcinoma was the indication for 84% of robotic and 63% of laparoscopic procedures. Perforation was the indication in 11 laparoscopic patients (11%) and no robotic patients. Emergency operations were uncommon in the robotic cohort (2%) but accounted for 20% of laparoscopic procedures (p < 0.001).

### Peri- and postoperative outcomes

**Fig. 3:**
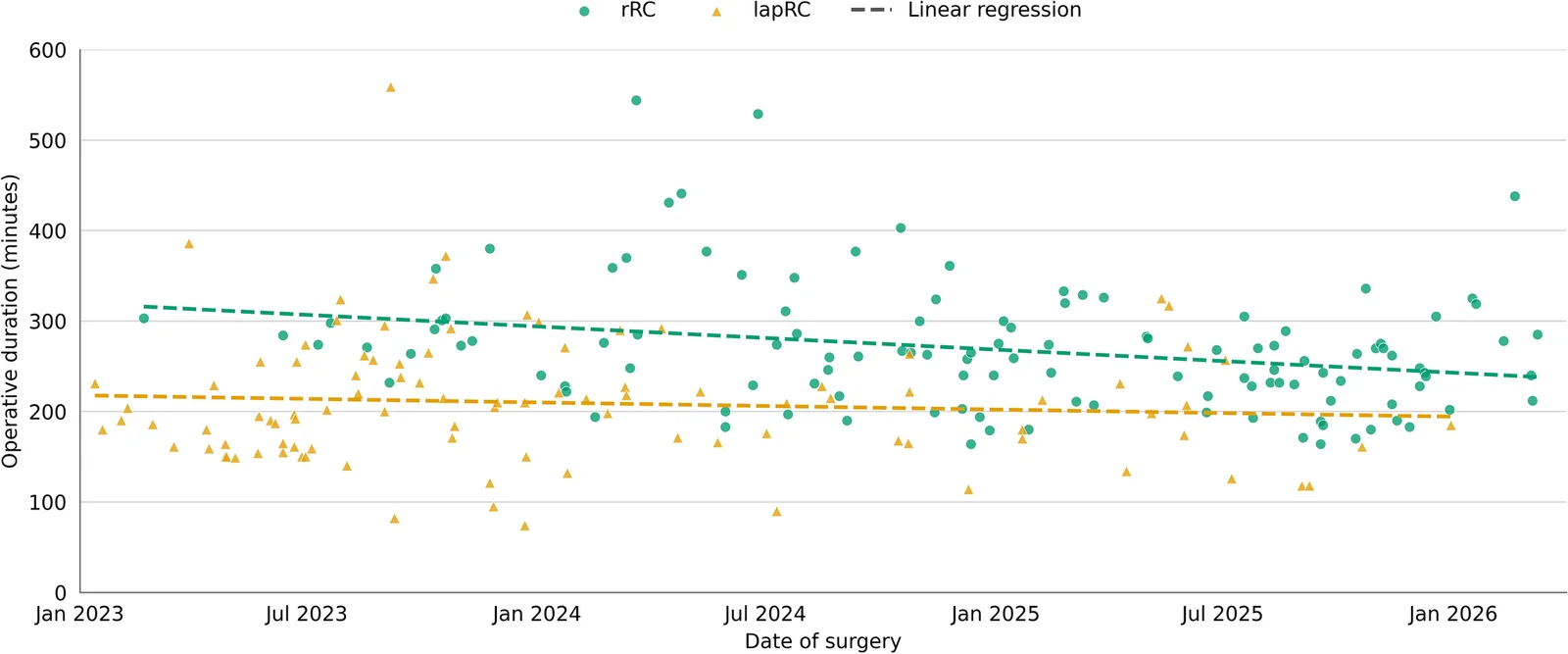
Operative duration over time for rRC and lapRC. Lines indicate unadjusted linear regression

Table 2 summarizes perioperative and postoperative outcomes. Procedures were performed by 14 surgeons in the rRC cohort and 29 surgeons in the lapRC cohort. Median procedure duration was 264 min (IQR 228–300) for rRC and 200 min (IQR 163–247) for lapRC (p < 0.001). The distribution of operative times is illustrated in Fig. 3. Procedure duration showed considerable variability in both cohorts, with higher median values and more high-duration observations in the robotic group. In unadjusted linear regression, robotic operative duration decreased by 25.5 min per calendar year (95% CI, 10.3–40.8 min shorter; p = 0.001). No significant temporal trend was found for lapRC (p = 0.366)**.**

**Table 2:** Perioperative and 30-day postoperative outcomes

	rRC n=122	lapRC n=99	p-value
Operative duration, min, median (IQR)	264 (228–300)	200 (163–247)	<0.001
Type of resection, n (%) Right hemicolectomy Extended right hemicol	107 (88) 15 (12)	92 (93) 7 (7)	0.260
Teaching procedures, n (%)	32 (26)	31 (31)	0.455
Conversion to open surgery, n (%)	5 (4)	20 (20)	<0.001
Reason for conversion (n,%)^1^ Adhesions Tumor-related difficulty Bowel necrosis Unclear Perforation Ileus Obesity Abnormal anatomy Bleeding	0 (0) 1 (20) 0 (0) 0 (0) 2 (40) 0 (0) 1 (20) 1 (20)	6 (30) 8 (40) 4 (20) 1 (5) 0 (0) 1 (5) 0 (0) 0 (0)	-
Stoma creation, n (%)	0 (0)	2 (2)	0.200
Hospital stay, days, median (IQR)	6 (5–8)	6 (5–9)	0.207
Any postoperative complication, n (%)	34 (28)	35 (35)	0.246
Clavien-Dindo classification, n (%)^2^ No complication CD I CD II CD IIIa CD IIIb CD IVa CD IVb CD V	88 (72) 6 (5) 13 (11) 2 (2) 4 (3) 7 (6) 1 (1) 1 (1)	64 (65) 3 (3) 20 (20) 6 (6) 1 (1) 2 (2) 3 (3) 0 (0)	0.072
Major complications, CD ≥III, (%) Bleeding Anastomotic leak Wound dehiscence Priapism Small bowel fistula Cardiac complication Ileus requiring reoperation Intra-abdominal abscess Incisional hernia Sepsis C. difficile infection	15 (12) 2 (2) 1 (1) 0 (0) 0 (0) 0 (0) 5 (4) 2 (2) 1 (1) 2 (2) 1 (1) 1 (1)	12 (12) 4 (4) 4 (4) 1 (1) 1 (1) 1 (1) 1 (1) 0 (0) 0 (0) 0 (0) 0 (0) 0 (0)	1.000 - 0.176 - - - - - - - - -
CCI, median (IQR)	0 (0–8.7)	0 (0–20.9)	0.307
30-day reoperation, n (%)	4 (3)	6 (6)	0.349
30-day readmission, n (%)	3 (3)	0 (0)	0.255
30-day mortality, n (%)	1 (1)	0 (0)	1.000

Values are median (IQR) or n (%). ^1^Percentages refer to converted patients. ^2^Overall comparison including patients without complications. CD, Clavien–Dindo; CCI, Comprehensive Complication Index; IQR, interquartile range; rRC, robotic right hemicolectomy; lapRC, laparoscopic right hemicolectomy

Open conversion was required in 5 patients (4%) undergoing rRC and in 20 patients (20%) undergoing lapRC (p < 0.001). The recorded reasons for laparoscopic conversion were tumor size (n = 8), adhesions (n = 6), bowel necrosis (n = 4), unclear perforation (n = 1), and obesity (n = 1). Robotic conversions were required for ileus with difficult exposure or anatomical orientation in two patients, intestinal malrotation with unclear dissection planes in one, bleeding from an ileocolic vessel branch that could not be controlled minimally invasively in one, and tumor-related mesenteric retraction with suspected central vascular involvement in one. In all five cases, conversion preceded anastomosis formation, and reconstruction was performed openly. A prophylactic stoma was created in two lapRC patients and in none of the rRC patients (p = 0.200).

Hospital stay was identical in median duration between groups, at 6 days for both rRC and lapRC (IQR 5–8 and 5–9 days, respectively; p = 0.207). Any postoperative complication was recorded in 28% of robotic and 35% of laparoscopic cases (p = 0.246). Major morbidity, corresponding to Clavien–Dindo grade III or higher, occurred in 12% of each cohort (p = 1). Median CCI values were 0 (IQR 0–8.7) for rRC and 0 (IQR 0–20.9) for lapRC (p = 0.307).

Anastomotic leakage occurred in 1% of rRC and 4% of lapRC patients (p = 0.176). Within 30 days, reoperation was necessary in 3% and 6% of patients, respectively (p = 0.349). Rehospitalization during the same period occurred in three patients after rRC and in no patients after lapRC (p = 0.255). One death within 30 days of surgery occurred in the robotic cohort and none in the laparoscopic cohort (p = 1).

### Oncological outcomes

The oncological findings are summarized in Table 3. Among the 111 oncological procedures in the rRC cohort and 66 in the lapRC cohort, carcinoma was diagnosed in 103 (93%) and 62 (94%) patients, respectively (p = 1). The remaining cases were neuroendocrine tumors. The following carcinoma-specific pathological analyses excluded neuroendocrine tumors.

**Table 3:** Oncological and pathological findings

**A. All oncological resections**			
	rRC n=111	lapRC n=66	p-value
Tumor type, n (%) Carcinoma NET	103 (93) 8 (7)	62 (94) 4 (6)	1.000
Tumor location, n (%) Appendix Cecum Ascending colon Hepatic flexure Transverse colon Splenic flexure Other (small intestine)	4 (4) 42 (38) 37 (33) 9 (8) 13 (12) 1 (1) 5 (5)	3 (5) 23 (35) 25 (38) 10 (15) 3 (5) 0 (0) 2 (3)	0.482
**B. Carcinoma-specific findings**			
	rRC n=103	lapRC n=62	p-value
Documented clinical T-stage, n cT1, n (%)^1^ cT2 cT3 cT4 Tx or undocumented	34 3 (9) 5 (15) 13 (38) 13 (38) 69	16 1 (6) 3 (19) 9 (56) 3 (19) 46	0.521 -
Pathological T-stage, n (%) pT0 pT1 pT2 pT3 pT4	13 (13) 16 (16) 16 (16) 36 (35) 22 (21)	2 (3) 6 (10) 13 (21) 32 (52) 9 (15)	0.063
Pathological N-stage, n (%) pN0 pN1 pN2	73 (71) 17 (17) 13 (13)	38 (61) 16 (26) 8 (13)	0.339
Recorded M-status, n (%)^2^ M0 M1 Undocumented	97 (94) 6 (6) 0 (0)	58 (94) 3 (5) 1 (2)	1.000
Documented histopathological grade, n G1, n (%)^1^ G2 G3 Undocumented grade, n	101 39 (39) 48 (48) 14 (14) 2	61 7 (11) 43 (70) 11 (18) 1	<0.001 -
R-status, n (%) R0 R1	102 (99) 1 (1)	62 (100) 0 (0)	1.000
Retrieved lymph nodes, median (IQR)	26 (20.5–32)	25 (18–31)	0.249
≥12 retrieved lymph nodes, n (%)	102 (99)	61 (98)	1.000
Qualitative CME assessment, n (%) Good quality documented Undocumented	16 (16) 87 (84)	13 (21) 49 (79)	-

Values are n (%) unless otherwise indicated. ^1^Percentages refer to cases with a documented category. ^2^The comparison excludes undocumented M-status. P values refer to overall distributions. CME assessments were qualitative and did not represent standardized mesocolic-plane grading. CME, complete mesocolic excision; IQR, interquartile range; NET, neuroendocrine tumor; rRC, robotic right hemicolectomy; lapRC, laparoscopic right hemicolectomy

The median number of retrieved lymph nodes was 26 (IQR 20.5–32) after rRC and 25 (IQR 18–31) after lapRC, without evidence of a difference between approaches (p = 0.249). At least 12 lymph nodes were retrieved in 102/103 (99%) and 61/62 (98%) carcinoma resections, respectively. An R0 resection was obtained in 102/103 (99%) robotic and 62/62 (100%) laparoscopic carcinoma resections (p = 1).

The overall pathological T-stage distribution did not differ significantly between groups (p = 0.063). pT3 tumors accounted for 35% of robotic and 52% of laparoscopic carcinoma cases, while pT4 tumors accounted for 21% and 15%, respectively. pT0 was recorded in 13 robotic and two laparoscopic cases following completion colectomy after endoscopic removal of a histologically confirmed carcinoma, with no residual primary tumor in the surgical specimen. Among cases with documented grading, G1 tumors accounted for 39% and 11%, and G2 tumors for 48% and 70%, respectively; the overall grade distribution differed between groups (p < 0.001).

A qualitative assessment of good CME quality was documented in 16/103 robotic and 13/62 laparoscopic carcinoma resections. CME quality was undocumented in the remaining cases. These assessments did not represent standardized mesocolic-plane grading.

### Sensitivity analysis

The elective cohort comprised 120 rRC and 79 lapRC procedures. Median operative duration remained longer after rRC (264 vs. 214 min; p < 0.001). Conversion occurred in 4/120 (3.3%) robotic and 8/79 (10.1%) laparoscopic procedures (p = 0.067). Overall morbidity (27.5% vs. 32.9%; p = 0.431) and major morbidity (11.7% vs. 8.9%; p = 0.640) did not differ significantly.

Among elective oncological resections, including carcinomas and neuroendocrine tumors, 110 rRC and 64 lapRC procedures were analyzed. Median operative duration was longer after rRC (262 vs. 220 min; p < 0.001), while conversion occurred less frequently (3.6% vs. 12.5%; p = 0.033). Overall morbidity (27.3% vs. 32.8%; p = 0.491) and major morbidity (10.9% in both groups; p = 1.000) did not differ significantly. Full results are presented in Supplementary Table S1.

### Propensity-score-matched analysis

Propensity-score matching within the elective cohort yielded 32 pairs (64 patients). All matching covariates had absolute standardized differences below 0.10 after matching. Median operative duration remained longer after rRC (273 vs. 217 min; p = 0.003). Conversion occurred in one robotic (3.1%) and five laparoscopic patients (15.6%; p = 0.219). Overall morbidity (28.1% vs. 31.3%; p = 1.000) and major morbidity (18.8% vs. 12.5%; p = 0.727) did not differ significantly. Matched patient characteristics and outcomes are presented in Supplementary Table S2.

## Discussion

Robotic right hemicolectomy was associated with fewer conversions but longer operative duration, without statistically significant differences in short-term morbidity, resection margin status, or lymph-node yield. Longer operative duration persisted in supplementary analyses. The conversion difference remained significant in the main analysis and among elective oncological resections, but not in the overall elective or propensity-score-matched cohorts. Baseline imbalances and the small matched cohort limit interpretation. Increasing robotic use coincided with fewer primary open procedures, but the increase in overall minimally invasive completion was not statistically significant.

Among patients initially treated laparoscopically, tumor size, adhesions, and bowel necrosis were common recorded reasons for conversion; these were not evaluated as independent risk factors. The conversion rate was 4% after robotic surgery compared with 20% after laparoscopy (p < 0.001). However, emergency procedures were more frequent in the laparoscopic cohort (20% vs. 2%), and all 11 perforation cases were treated laparoscopically. These differences may explain part of the observed conversion difference. After excluding emergencies, the laparoscopic conversion rate was 10.1%. Robotic technology may also contribute to operative feasibility. Articulated instrumentation, three-dimensional high-definition imaging, surgeon-controlled visualization, tremor reduction, and improved ergonomics may facilitate dissection when exposure or tissue handling is difficult [14]. These features could be useful during technically demanding right hemicolectomy, including central vascular dissection for CME [15, 16]. The consistently lower conversion rates across the main, elective, and matched analyses suggest a potential advantage of robotic surgery in maintaining a minimally invasive approach. However, the small number of events and residual confounding prevent firm conclusions about an independent effect of the robotic platform.

Median operative duration was longer after rRC (264 vs. 200 min), consistent with previous reports [17–21]. Although experience may reduce this difference [22, 23], longer robotic operating times remain commonly reported [20, 21, 24–27]. In our cohort, robotic operative duration decreased by 25.5 min per calendar year, with no significant trend for laparoscopy. This unadjusted association may reflect learning, workflow refinement, or case selection. Docking was included but not recorded separately, and differences in anastomotic technique may also contribute to the time difference. Longer operative duration should be considered alongside the potentially lower conversion rate, as open conversion has been associated with complications, prolonged hospitalization, and higher costs [28–31].

Costs were not available for this study, precluding a direct economic comparison. The 64-min difference in median operative duration indicates greater operating room time requirements for robotic procedures and may affect staffing costs and theatre capacity. Merola et al. reported higher total costs for robotic than laparoscopic right colectomy, mainly reflecting instrument and operating room costs [32].

Postoperative morbidity did not differ significantly between approaches. Overall complications occurred in 28% of patients after rRC and 35% after lapRC (p = 0.246), while major complications occurred in 12% of both cohorts (p = 1). Anastomotic leakage was observed in 1% and 4%, respectively (p = 0.176). The median CCI was 0 (IQR 0–8.7) following rRC and 0 (IQR 0–20.9) following lapRC (p = 0.307). Because the absolute number of events was small, the study had limited ability to identify differences in uncommon outcomes such as anastomotic leakage or reoperation; both were numerically more frequent after laparoscopy. Conversely, CD IVa complications (6% vs. 2%), cardiac complications (4% vs. 1%), and reoperation for ileus (two vs. no patients) were numerically more frequent after robotic surgery. Cardiac events comprised three arrhythmias and two episodes of decompensation in patients with pre-existing coronary disease and pulmonary complications. Laparoscopy for ileus identified adhesive obstruction in one patient and no mechanical cause in the other. The small event counts and baseline imbalances limit conclusions about a platform-specific effect.

These findings agree with earlier comparative studies showing no major short-term safety differences between robotic and laparoscopic right colectomy [17, 18, 24, 33, 34]. Wang et al. similarly found no statistically significant differences in Clavien–Dindo complication rates or conversion rates in their meta-analysis of older patients undergoing colorectal cancer surgery [35]. This is relevant to the age profile of our cohort, although their analysis included both colon and rectal resections.

Resection margin status and lymph-node yield did not differ significantly between approaches. An R0 resection was obtained in 99% of patients after rRC and 100% after lapRC. Median lymph-node yield was 26 and 25, respectively (p = 0.249), with at least 12 nodes retrieved in 99% and 98% of patients. The higher proportions of pT0 cases and G1 tumors in the robotic cohort suggest differences in case selection that may influence interpretation of the pathological outcomes. Although documented clinical T-stage did not differ significantly between groups, its limited availability prevents firm conclusions about preoperative comparability. These findings are consistent with a multicentre propensity-score-matched study of CME, which reported comparable lymph-node yields and similar 2-year disease-free and overall survival after robotic and laparoscopic surgery [19]. However, lymph-node yield alone does not establish CME quality, and standardized mesocolic-plane grading and specimen length were unavailable in our study.

The oncological value of extensive mesocolic dissection remains uncertain, since the phase III RELARC trial found no significant difference in 3-year disease-free or overall survival between laparoscopic CME and D2 dissection, while vascular injuries were more frequent with CME [5, 6]. Five-year follow-up likewise showed no significant overall survival benefit, although subgroup analyses suggested a benefit in patients with pathological N2 disease [36]. The enhanced visualization and instrument mobility available with robotic surgery could potentially facilitate complex central vascular dissection. However, our study did not directly assess this technical advantage or long-term oncological outcomes.

At present, prospective long-term oncological evidence specifically for robotic right hemicolectomy remains limited. The randomized phase II RoLaCaRT-1 pilot trial reported no significant differences in pathological specimen quality and lower short-term morbidity after robotic surgery, but included only 29 patients and could not establish long-term oncological superiority [7]. Larger randomized studies with adequate follow-up are needed to determine whether these short-term findings translate into lasting clinical benefits.

In the full screened series, increasing robotic use coincided with fewer primary open procedures. However, the increase in overall minimally invasive completion did not reach statistical significance. As robotic surgery had been introduced in 2015, our study describes changes within an established programme rather than its initial implementation. These temporal associations do not establish that robotic adoption caused the reduction in open surgery.

### Limitations

This study has several limitations. Its retrospective single-centre design may have introduced selection bias and residual confounding. The choice of surgical approach was not randomised and depended on surgeon preference, availability of the robotic platform, and the clinical situation. Emergency procedures and perforation were more frequent in the laparoscopic cohort, limiting interpretation of the unadjusted comparison. Furthermore, robotic procedures completed without open conversion used intracorporeal anastomoses, whereas laparoscopic procedures used extracorporeal anastomoses. Differences in outcomes therefore cannot be attributed to the surgical platform alone.

Sensitivity analyses restricted to elective procedures and propensity-score matching addressed some baseline differences, but residual confounding remains possible. The matched analysis included only 32 pairs and few outcome events, limiting statistical power and generalisability. Clinical T-stage could not be included in matching because of limited availability. These exploratory analyses cannot establish equivalence between approaches or an independent effect of the robotic platform**.**

Although robotic surgery was introduced at our institution in 2015, increasing experience and workflow refinement may still have influenced outcomes during the study period. The observed reduction in robotic operative duration was based on unadjusted analysis and may also reflect changes in case selection. Individual surgeon learning curves were not assessed.

Postoperative outcomes were assessed only within 30 days using institutional records; events treated elsewhere may have been missed. This follow-up was insufficient to assess long-term incisional hernia rates or oncological outcomes. Cost data were unavailable, preventing an assessment of cost-effectiveness.

## Conclusion

In this retrospective cohort, robotic right hemicolectomy was associated with fewer conversions but longer operative duration than laparoscopy. No statistically significant differences were found in short-term morbidity, resection margin status, or lymph-node yield. The lower conversion rates suggest a potential benefit of robotic surgery, but baseline imbalances and the small matched cohort limit conclusions about an independent platform effect. Larger prospective studies are needed to confirm this potential benefit.

## Supplementary Information

Below is the link to the electronic supplementary material.

Supplementary file1

## Data Availability

The data analysed during the current study are available from the corresponding author.
