# Supplementary material for: Robotic versus laparoscopic right hemicolectomy: a retrospective single-center comparative cohort study
Source: J Robot Surg. 2026 Oct 26;20(1):1137. doi: 10.1007/s11701-026-04070-5 (PMC13696575; doi:10.1007/s11701-026-04070-5)
Supplement: Supplementary file 1 [file 11701_2026_4070_MOESM1_ESM.docx]

**Supplementary material**

***Table S1*** *Outcomes in elective and elective oncological resections*

| A. **All elective procedures** | | | |
| --- | --- | --- | --- |
|  | rRC  n=120 | lapRC  n=79 | p-value |
| Operative duration, min, median (IQR) | 264 (228-299) | 214 (180-257) | <0.001 |
| Conversion to open surgery, n (%) | 4 (3) | 8 (10) | 0.067 |
| Any postoperative complications, n (%) | 33 (28) | 26 (33) | 0.431 |
| Major morbidity, CD ≥III, n (%) | 14 (12) | 7 (9) | 0.640 |
| Anastomotic leak, n (%) | 1 (1) | 3 (4) | 0.303 |
| Hospital stay, days, median (IQR) | 6 (5-8) | 6 (5-8.5) | 0.637 |
| CCI, median (IQR) | 0 (0-8.7) | 0 (0-20.9) | 0.565 |
| 30-day reoperation, n (%) | 4 (3) | 3 (4) | 1.000 |
| 30-day readmission, n (%) | 3 (3) | 0 (0) | 0.278 |
| 30-day mortality, n (%) | 0 (0) | 0 (0) | - |
| B. **Elective oncological outcomes** | | | |
|  | rRC  n=110 | lapRC  n=64 | p-value |
| Operative duration, min, median (IQR) | 262 (224-293) | 220 (184-258) | <0.001 |
| Conversion to open surgery, n (%) | 4 (4) | 8 (13) | 0.033 |
| Any postoperative complications, n (%) | 30 (27) | 21 (33) | 0.491 |
| Major morbidity, CD ≥III, n (%) | 12 (11) | 7 (11) | 1.000 |
| Anastomotic leak, n (%) | 1 (1) | 3 (5) | 0.141 |
| Hospital stay, days, median (IQR) | 6 (5-8) | 6 (5-8) | 0.642 |
| CCI, median (IQR) | 0 (0-8.7) | 0 (0-20.9) | 0.587 |
| 30-day reoperation, n (%) | 3 (3) | 3 (5) | 0.671 |
| 30-day readmission, n (%) | 3 (3) | 0 (0) | 0.298 |
| 30-day mortality, n (%) | 0 (0) | 0 (0) | - |

*Values are median (IQR) or n (%). Operative duration is rounded to whole minutes. Oncological resections include carcinomas and neuroendocrine tumors. All postoperative outcomes refer to 30 days. No p value is reported for mortality because neither group had an event. CD, Clavien–Dindo; CCI, Comprehensive Complication Index; IQR, interquartile range; rRC, robotic right hemicolectomy; lapRC, laparoscopic right hemicolectomy.*

***Table S2*** *Patient characteristics and outcomes after propensity-score matching*

| A. **Patient characteristics** | | | |
| --- | --- | --- | --- |
|  | rRC  n=32 | lapRC  n=32 | Absolute SMD |
| Age, years, median (IQR) | 71.0 (62.8-80.3) | 73.5 (68.8-77.8) | 0.044 |
| BMI, kg/m², median (IQR) | 24.3 (22.2–29.8) | 24.5 (22.5–27.5) | 0.067 |
| Sex, n (%)  Male  Female | 19 (59)  13 (41) | 19 (59)  13 (41) | 0.000 |
| ASA group, n (%)  I/II  III  IV | 6 (19)  24 (75)  2 (6) | 6 (19)  24 (75)  2 (6) | 0.000  0.000  0.000 |
| Surgical indication, n (%)  Carcinoma  NET  Other | 25 (78)  0 (0)  7 (22) | 25 (78)  0 (0)  7 (22) | 0.000  0.000  0.000 |
| *Values are median (IQR) or n (%). Absolute standardized differences use the unmatched elective cohort’s standardizing denominator; values <0.10 indicate adequate balance. NET cases were eligible for matching, but none were retained in the matched cohort.* | | | |
| B. **Matched outcomes** | | | |
|  | rRC  n=32 | lapRC  n=32 | p-value |
| Operative duration, min, median (IQR) | 273 (245-293) | 217 (182-238) | 0.003 |
| Conversion to open surgery, n (%) | 1 (3) | 5 (16) | 0.219 |
| Any postoperative complications, n (%) | 9 (28) | 10 (31) | 1.000 |
| Major morbidity, CD ≥III, n (%) | 6 (19) | 4 (13) | 0.727 |
| Anastomotic leak, n (%) | 1 (3) | 2 (6) | 1.000 |
| Hospital stay, days, median (IQR) | 6 (5-7) | 6 (5-9.25) | 0.324 |
| CCI, median (IQR) | 0 (0-8.7) | 0 (0-20.9) | 0.856 |
| 30-day reoperation, n (%) | 3 (9) | 2 (6) | 1.000 |
| 30-day readmission, n (%) | 2 (6) | 0 (0) | 0.500 |
| 30-day mortality, n (%) | 0 (0) | 0 (0) | - |
| *Values are median (IQR) or n (%). Operative duration is rounded to whole minutes. Binary outcomes were compared using exact McNemar tests and continuous outcomes using Wilcoxon signed-rank tests. All postoperative outcomes refer to 30 days. No p value is reported for mortality because neither group had an event.* | | | |

*ASA, American Society of Anesthesiologists; BMI, body mass index; CD, Clavien–Dindo; CCI, Comprehensive Complication Index; IQR, interquartile range; NET, neuroendocrine tumor; SMD, standardized mean difference; rRC, robotic right hemicolectomy; lapRC, laparoscopic right hemicolectomy.*
